# Newly discovered mechanisms that mediate tumorigenesis and tumour progression: circRNA‐encoded proteins

**DOI:** 10.1111/jcmm.17751

**Published:** 2023-04-18

**Authors:** Chengwei Wu, Song Wang, Tingting Cao, Tao Huang, Lishuai Xu, Jiawei Wang, Qian Li, Ye Wang, Long Qian, Li Xu, Yabin Xia, Xiaoxu Huang

**Affiliations:** ^1^ Department of Gastrointestinal Surgery The First Affiliated Yijishan Hospital of Wannan Medical College Wuhu China; ^2^ Key Laboratory of Non‐coding RNA Transformation Research of Anhui Higher Education Institution Wannan Medical College Wuhu China; ^3^ Department of Thoracic Surgery The First Affiliated Yijishan Hospital of Wannan Medical College Wuhu China; ^4^ The Second Affiliated Hospital of Wannan Medical College Wuhu China

**Keywords:** circRNA, circRNA‐encoded protein, research methods, translational medicine, tumour malignancy progression

## Abstract

Proteins produced by cap‐independent translation mediated by an internal ribosome entry site (IRES) in circular RNAs (circRNAs) play important roles in tumour progression. To date, numerous studies have been performed on circRNAs and the proteins they encode. In this review, we summarize the biogenesis of circRNAs and the mechanisms regulating circRNA‐encoded proteins expression. We also describe relevant research methods and their applications to biological processes such as tumour cell proliferation, metastasis, epithelial‐mesenchymal transition (EMT), apoptosis, autophagy and chemoresistance. This paper offers deeper insights into the roles that circRNA‐encoded proteins play in tumours. It also provides a theoretical basis for the use of circRNA‐encoded proteins as biomarkers of tumorigenesis and for the development of new targets for tumour therapy.

## 1 INTRODUCTION

In recent years, due to the rapid development and extensive application of high‐throughput RNA sequencing and new bioinformatics algorithms, a large number of circular RNAs (circRNAs) have been identified in the cells of many eukaryotes. circRNAs are novel RNA molecules that are produced from precursor mRNAs by reverse splicing, which differs from the production of conventional linear RNAs. This unconventional process results in a closed‐loop RNA structure with neither a 3′ poly(A) tail nor a 5′ cap structure, rendering circRNAs more stable and resistant to ribonuclease degradation than linear RNAs. Over the years, considerable research has been reported on the general functions of circRNAs: they can act as endogenous competitive RNAs such as microRNA (miRNA) sponges, they can interact with RNA‐binding proteins (RBPs), they can regulate the transcription of parental genes, and they can form short double‐stranded RNAs to suppress protein kinase R (PKR).^1^, ^2^, ^3^ It is generally accepted that circRNAs constitute a regulatory noncoding RNA subtype and usually do not encode proteins, but perform important functions such as regulating transcription, RNA splicing modification, mRNA translation, and protein stabilization and translocation. They also play roles in chromosome formation and structural stability.^4^ Recently, bioinformatics analysis has suggested that circRNAs show the potential to encode proteins and can perform biological functions by encoding certain proteins. Most circRNAs consist of exonic sequences, localize to the cytoplasm and carry open reading frames (ORFs) with start codons, which indicate their potential to encode proteins.^5^ The current view suggests that cap‐independent translation of circRNAs that encode proteins is typically initiated through internal ribosome entry sites (IRESs) or N6‐methyladenosine (m6A)‐induced ribosome engagement sites (MIRESs). While most studies on tumorigenesis and tumour progression have focused generally on linear parental gene changes and have neglected circRNAs, recent studies have identified and elucidated multiple circRNA‐encoded proteins that play important roles in human tumorigenesis and progression through their respective mechanisms. In this review, we systematically describe the biogenesis of circRNAs, the mechanisms regulating circRNA‐encoded protein expression, relevant research methods and their roles in biological processes in tumours. We also look forward to the application of circRNA‐encoded proteins in treatment and raise the issue of the limitations of the current studies that need to be addressed. This paper provides a theoretical basis for future clinical applications and guides future research on circRNA‐encoded proteins.

## 2 BIOGENESIS OF circRNAs

**FIGURE 1:**
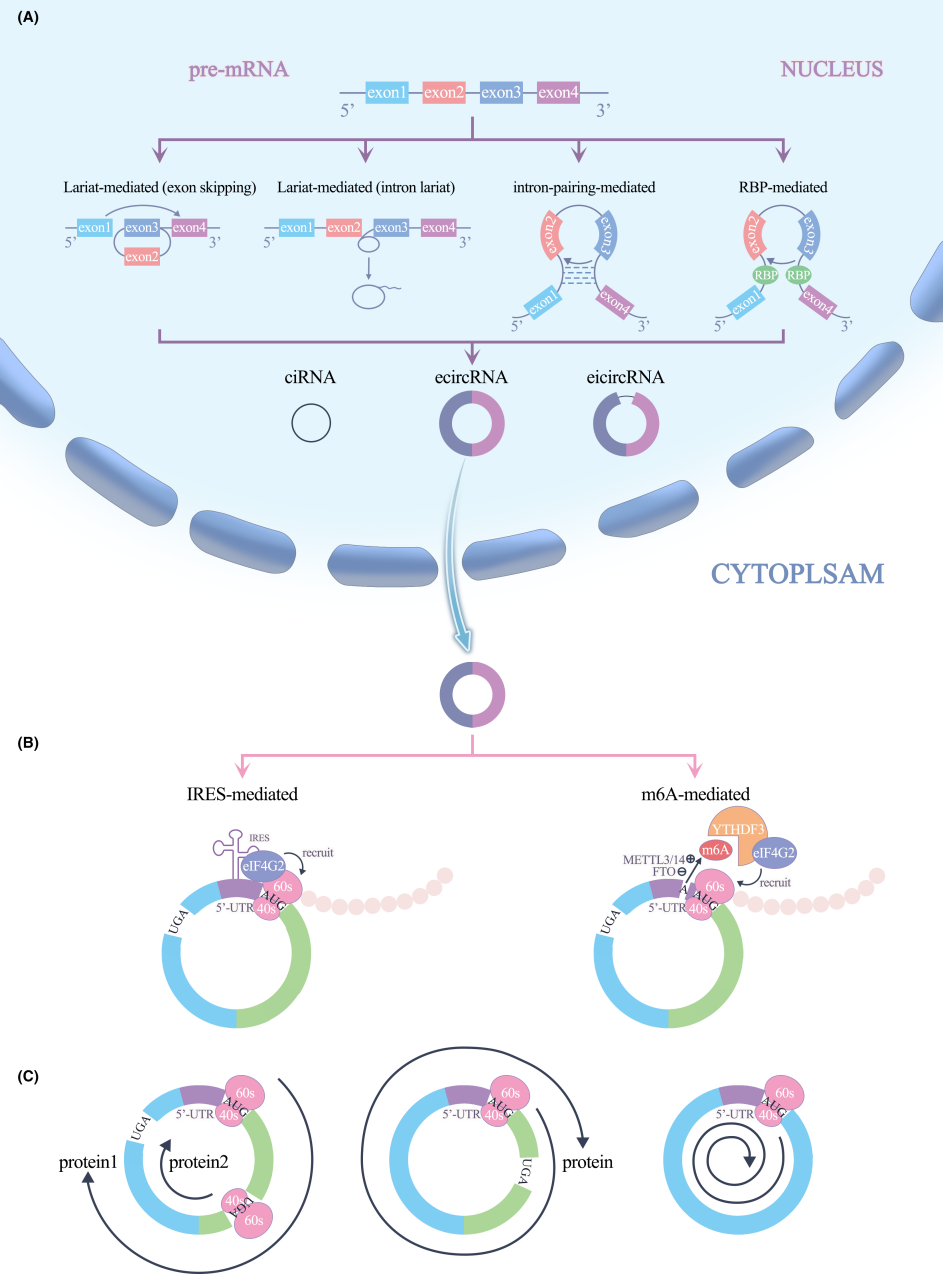
(A) Biogenesis of circRNAs. (B) IRES/MIRES driven translation. (C) Three types of translation.

Most circRNAs are formed from exons or introns by back‐splicing of pre‐mRNA, and they compete with linear mRNAs during splicing. CircRNAs are largely classified into three types on the basis of their source RNA sequence and method of circularization: exonic circRNAs (ecircRNAs), intronic circRNAs (ciRNAs) and exon–intron circRNAs (eicircRNAs), ecircRNAs are the most abundant. It is generally accepted that circRNAs are circularized by the following four mechanisms. In the exon‐skipping mechanism, exons skip during partial RNA folding during pre‐mRNA transcription, and these structural changes lead to the formation of specific regions called lariat structures. A ‘hetero‐lariat’ is first formed and contains exons and introns, so this type of circRNA is called eicircRNA. Removing the introns in eicircRNA can generate ecircRNA containing only exons.^2^, ^6^, ^7^, ^8^ In the intron lariat circularization mechanism, some introns are thought to form lariat structures during splicing, but most introns are rapidly degraded by debranching, with only some introns containing essential nucleic acid sequences that remain branched after splicing.^9^ Pre‐mRNA is cleaved at the 5′ end, a process facilitated by small nuclear RNA (snRNA) U1, near the intron lariat. Then, the cleaved RNA is further processed to form a ciRNA.^10^ Base pairing of the flanking intron sequence drives circularization mainly by pairing introns between exons (Alu repeat sequence) to induce reverse splicing, and the introns covalently bind together to form circRNA. RBP‐mediated intron pairing drives circularization by introns flanking of the exons in which the RBP binds to the pre‐mRNA. RBP dimerization promotes the cyclization of adjacent exons to form circRNA. These two mechanisms can generate all three types of circRNAs^11^, ^12^, ^13^, ^14^ (Figure 1A).

## 3 MECHANISM OF circRNA‐ENCODED PROTEINS

Translation of RNA in eukaryotic cells requires the eukaryotic translation initiation factor eIF4F, a complex consisting of the decapping enzyme eIF4A, cap‐binding subunit eIF4E and scaffolding protein eIF4G. eIF4G recruits the 43S pre‐initiation complex (PIC), which includes the 40S small ribosomal subunit, eIF1, eIF1A, eIF3, eIF5 and the eIF2/Met‐tRNAi/GTP ternary complex by interacting with eIF3.^15^, ^16^ Upon recognizing the 5′‐end cap structure of mRNA, eIF4F recruits the 43S PIC complex, enabling the translation process.^17^ This canonical translation initiation mechanism is called cap‐dependent translation initiation and is the main mechanism of translation initiation in eukaryotic cells.^17^, ^18^ CircRNAs follow a noncanonical cap‐independent translation mechanism due to the lack of a 5′ cap structure and depend on IRESs or MIRESs to bind to the initiation factor eIF4G2 complex and anchor the 43S complex for protein translation (Figure 1B). Interestingly, under cell stress conditions, such as hypoxia, amino‐acid starvation, endoplasmic reticulum (ER) stress and apoptosis, as well as after viral infection and exposure to physiological cell differentiation or synaptic network formation stimuli, this translation mechanism is initiated as an alternative to that of naturally endogenous mRNA translation mechanisms.^19^, ^20^

### 3.1 Internal ribosome entry sites

IRESs are RNA elements that promote the recruitment of the 40S ribosomal subunit to the internal regions of mRNAs to initiate translation, and participate in translation initiation through a 5′‐cap‐independent mechanism.^21^ IRESs were first identified in the 5′‐untranslated region (5’‐UTR) of small RNA viruses detected in animal viral RNA.^22^ Specifically, an IRES is a 200‐ to 500‐nucleotide‐long sequence located in the 5’‐UTR with a specific stem‐loop structure that facilitates the translation of many pathogenic viruses. Later, studies revealed IRESs in eukaryotic mRNAs, and under stress conditions, IRES‐mediated cap‐independent translation can serve as an alternative mechanism for protein production from linear mRNAs in eukaryotic cells.^23^ Follow‐up studies have shown that circRNAs with IRESs can translate polypeptide chains from their ORFs. Interestingly, due to the special structure of circRNAs, an IRES sequence in circRNA may be read multiple times; that is in the first read, the IRES sequence is recognized, and in the following reads, the IRES encoding information is translated. Multiple reads result in multiple rounds of translation, which is very common for circRNA‐encoded protein production (Figure 1C).

### 3.2 N6‐methyladenosine (m6A) modification

m6A is a methylation on the sixth nitrogen element of the adenosine base in eukaryotic RNA and is the most prevalent, abundant and dynamically reversible episodic transcriptome modification in mammals.^24^ The m6A modification process relies on a recognized motif sequence ‘RRm6ACH’ (R = G or A; H = A, C or U).^25^ The m6A modification is regulated by m6A methyltransferases, m6A demethyltransferases and m6A‐binding proteins. Because abnormalities in m6A regulatory mechanisms can lead to the dysregulation of gene expression, including the activation and repression of oncogenes, they are often associated with tumour progression and play important roles in malignant progression and the acquisition of drug resistance in various types of tumours.^26^, ^27^ In addition, m6A plays a regulatory role in the translation of RNA. As an IRES, a MIRES stimulates selective mRNA translation under stress conditions by directly binding to the initiation factor eIF3. For circRNAs, m6A modification regulates not only the expression, distribution and function of circRNAs but also the translation of circRNAs. Many translatable endogenous circRNAs carry m6A modification sites, and MIRESs have been reported to act as IRES‐like elements to drive the translation of circRNAs.^28^, ^29^ CircRNAs are efficiently translated via 19‐nucleotide short consensus sequences (RRm6ACH) carrying m6A sites.^30^ The m6A‐binding protein YTHDF3 recognizes m6A and recruits eIF4G2 to the m6A site. eIF4G2 recognizes an IRES and initiates the assembly of the eIF4 complex, recruiting ribosomes and initiating translation. Moreover, a single m6A site is sufficient to initiate cap‐independent translation.^31^, ^32^

### 3.3 Rolling circle translation (RCT)

When a ribosome translates a protein encoded by a covalently closed circRNA and never encounters a stop codon within an ORF, multiple rounds of translation may occur until the ribosome encounters a stop codon within an ORF. Under certain circumstances, iRCT occurs. Specifically, when the ribosome reads the start codon to initiate translation without encountering the termination codon within an ORF in a circRNA, the result is an infinite open reading frame (iORF), which eventually leads to iRCT.^33^, ^34^ Theoretically, after this mode of translation has been initiated, extremely high‐molecular‐weight proteins are eventually produced. Thus, it seems that RCT may be a novel mechanism of circRNA‐encoded protein translation^35^ (Figure 1C).

## 4 RESEARCH METHODS TO DETERMINE WHETHER circRNAs ENCODE PROTEINS

### 4.1 Prediction of necessary coding factors

ORF prediction, IRES prediction and m6A prediction are methods of determining whether circRNAs have protein‐encoding capacity. An ORF is a nucleic acid sequence in RNA that begins at the AUG codon and continues through a series of three base sets to a stop codon. In addition, regulatory elements upstream of the ORF, that is, an IRES or a MIRES, mediate the initiation of translation. For example, ORF Finder and sORFs.org can be used to find possible ORFs in the complete circRNA sequence provided, IRESite and IRES Finder can predict potential IRES on the provided circRNA sequences, and integrated bioinformatics tools such as circPRO and circRNADB.^36^, ^37^, ^38^, ^39^, ^40^

### 4.2 Dual‐luciferase reporter assay and m6A analysis

To determine whether circRNAs are translated, the presence of ORFs and functional IRES‐like elements is can be confirmed with luciferase assays, while m6A‐like elements is can be confirmed by m6A analysis.^41^

### 4.3 Insertion of protein tags (Flags) in combination with western blotting (WB)

Overexpression vectors can be constructed by inserting a protein tag upstream of an ORF putative stop codon; the following experimental groups are typical: (i) no Flag tag, (ii) Flag tag, and (iii) Flag tag with a promoter mutation. Finally, in combination with WB, the presence of circRNA‐encoded protein can be verified. Theoretically, if this circRNA can be translated, WB can detect the protein in the Flag tag group but not the other groups, and this Flag tag can also be used to identify the RNAs or proteins that may interact with circRNA‐encoded proteins by RNA sequencing (RNA‐seq) or mass spectrometry (MS). Moreover, immunofluorescence assays can be performed to assess the subcellular localization of circRNA‐encoded proteins.^42^, ^43^, ^44^

## 5 ROLES AND MECHANISMS OF circRNA‐ENCODED PROTEINS IN TUMOURS

Many studies have identified many circRNA‐encoded proteins that are closely related to tumours. These circRNA‐encoded proteins are either upregulated or downregulated or the respective downstream signalling pathways are activated or specific molecules are modified, affecting the epigenetic function of tumour cells or their chemoresistance in a variety of ways, ultimately mediating tumorigenesis and tumour progression.

### 5.1 How circRNA‐encoded proteins regulate tumour cell proliferation and metastasis

**FIGURE 2:**
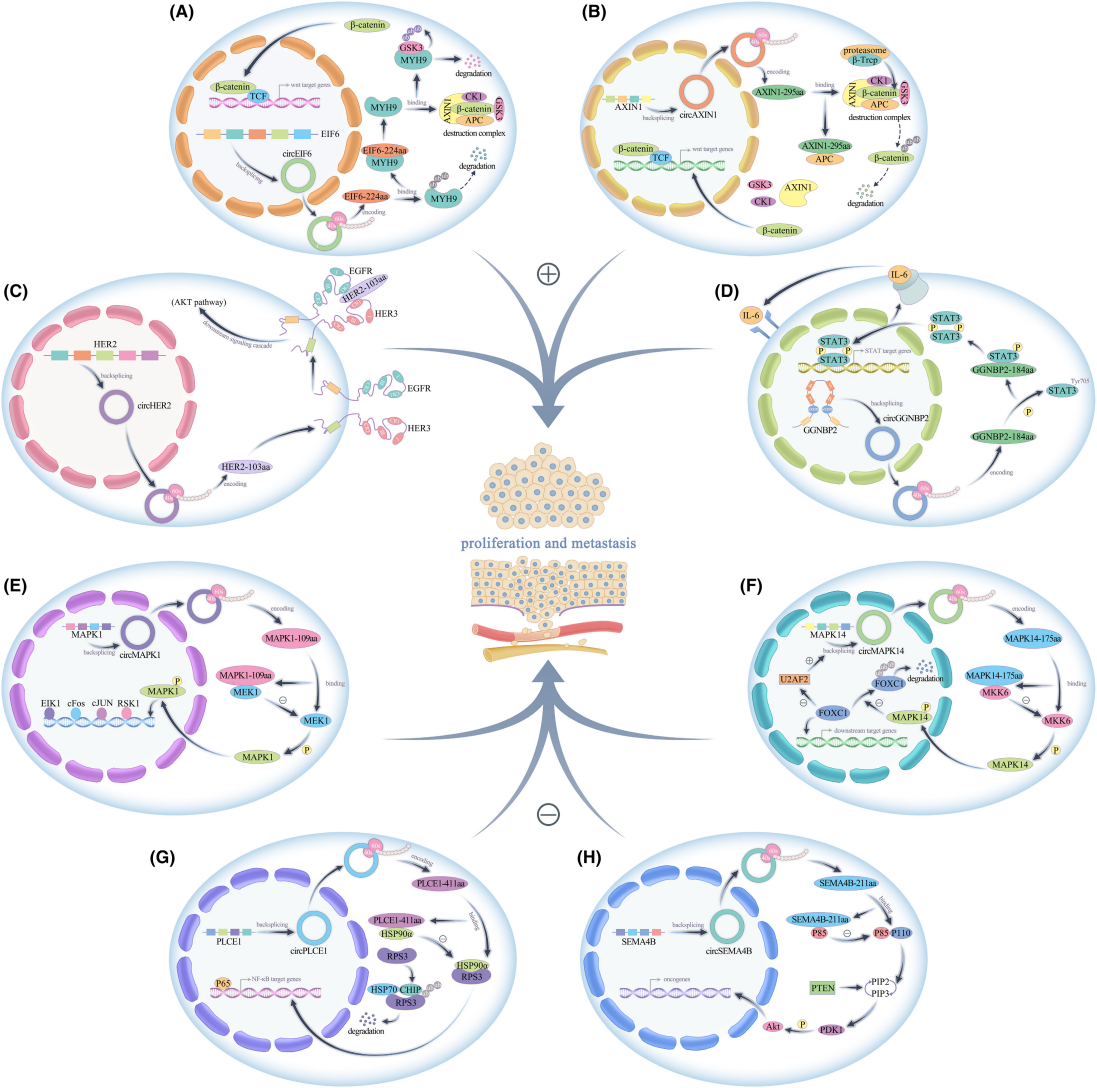
(A–D) circEIF6‐224aa, circAXIN1‐295aa, circHER2‐103aa, circGGNBP2‐184aa promote tumour proliferation and metastasis. (E–H) circMAPK1‐109aa, circMAPK14‐175aa, circPLCE1‐411aa, circSEMA4B‐211aa inhibit tumour proliferation and metastasis.

The upregulated expression of circRNA‐encoded proteins in tumour cells is important for tumour cell proliferation. Peng's team found that circAXIN1 expression was increased in gastric cancer tissues and promoted the proliferation and migration of gastric cancer cells by encoding the novel protein AXIN1‐295aa. Mechanistically, AXIN1‐295aa saturated available APC by competitively binding APC, resulting in the inability of AXIN1, CK1 or GSK3β to form a normal β‐catenin destruction complex with APC, leading to the translocation of β‐catenin to the nucleus where it activated downstream genes and ultimately promoted cell proliferation and migration.^45^ Li et al. found that circ‐EIF6 expression was increased in breast cancer tissues and that its encoded protein EIF6‐224aa inhibited its ubiquitin‐dependent degradation by interacting with the oncogene MYH9, thereby activating the MYH9/Wnt/β‐catenin pathway and ultimately promoting the proliferation and metastasis of triple‐negative breast cancer cells.^46^ Similarly, another study identified increased expression of circHER2 and its encoded protein HER2‐103aa specifically in HER2(‐) triple‐negative breast cancers. Mechanistically, HER2‐103aa induced the formation of epidermal growth factor receptor (EGFR)/EGFR homodimer and/or EGFR/HER3 heterodimer to maintain AKT phosphorylation, activate the downstream PI3K‐AKT pathway and promote triple‐negative breast cancer cell proliferation and progression.^47^ A recent study showed that circGGNBP2 expression was increased in intrahepatic cholangiocarcinoma tissues and promoted the proliferation and metastasis of intrahepatic cholangiocarcinoma cells by encoding the GGNBP2‐184aa protein. IL‐6 induced circGGNBP2 production, and circGGNBP2 biogenesis was also regulated by RBP DExH‐box helicase 9 (DHX9), which in turn was regulated by IL‐6. circGGNBP2 encodes a protein, GGNBP2‐184aa, that interacts with STAT3, phosphorylates the Tyr705 site of STAT3 and initiates transcription of STAT3 downstream target genes, ultimately leading to IL‐6/GGNBP2‐184aa/STAT3 initiation of a positive feedback loop that maintains constitutive activation of IL‐6/STAT3 signalling in intrahepatic cholangiocarcinoma cells, thereby promoting the progression of intrahepatic cholangiocarcinoma.^48^ To date, studies have shown that the circPPP1R12A‐encoded protein PPP1R12A‐73aa, circ_0006401‐encoded peptide circ_0006401, circCUX1‐encoded protein p113 isoform, circE‐cadherin‐encoded protein E‐cadherin variant, circEGFR‐encoded protein rolling‐translated EGFR, circβ‐catenin‐encoded protein β‐catenin‐370aa, circHNRNPU‐encoded protein HNRNPU‐603aa, circCHEK1‐encoded protein CHEK1‐246aa and circSMO‐encoded protein SMO‐193aa promote tumour progression. Moreover, their expression has been shown to be elevated in tumours (Table 1), (Figure 2A–D).

**TABLE 1:** Summary of circRNAs‐encoded proteins.

circRNAs	circRNAs‐encoded proteins	Tumour	Expression	Effects	Reference
circEIF6	EIF6‐224aa	Breast cancer	Up	Proliferation and metastasis	^46^
circAXIN1	AXIN1‐295aa	Gastric cancer	Up	Proliferation and metastasis	^45^
circGGNBP2	GGNBP2‐184aa	Intrahepatic cholangiocarcinoma	Up	Proliferation and metastasis	^48^
circPPP1R12A	PPP1R12A‐73aa	Colorectal cancer	Up	Proliferation and metastasis	^49^
circCUX1	p113 isoform	Neuroblastoma	Up	Proliferation and metastasis	^50^
circular E‐cadherin	E‐cadherin protein variant	Glioblastoma	Up	Proliferation and metastasis	^51^
circEGFR	rolling‐translated EGFR	Glioblastoma	Up	Proliferation and metastasis	^35^
circβ‐catenin	β‐catenin‐370aa	Non‐small‐cell lung cancer	Up	Proliferation and metastasis	^52^
circHNRNPU	HNRNPU‐603aa	Multiple myeloma	Up	Proliferation and metastasis	^53^
circCHEK1	CHEK1‐246aa	Multiple myeloma	Up	Proliferation and metastasis	^54^
circ_0006401	circ_0006401 peptide	Molorectal cancer	Up	Proliferation and metastasis	^55^
circSMO	SMO‐193aa	Glioblastoma	Up	Proliferation and metastasis	^56^
circHER2	HER2‐103aa	Breast cancer	Up	Proliferation and metastasis	^47^
circAKT3	AKT3‐174aa	Glioblastoma	Down	Proliferation and metastasis	^57^
circHEATR5B	HEATR5B‐881aa	Glioblastoma	Down	Proliferation and metastasis	^58^
circFBXW7	FBXW7‐185aa	Breast cancer	Down	Proliferation and metastasis	^59^
circMAPK1	MAPK1‐109aa	Gastric cancer	Down	Proliferation and metastasis	^60^
circMAPK14	MAPK14‐175aa	Colorectal cancer	Down	Proliferation and metastasis	^61^
circPINTexon2	PINT‐87aa	Glioblastoma	Down	Proliferation and metastasis	^43^
circPLCE1	PLCE1‐411	Colorectal cancer	Down	Proliferation and metastasis	^62^
circSHPRH	SHPRH‐146aa	Glioblastoma	Down	Proliferation and metastasis	^63^
circSEMA4B	SEMA4B‐211aa	Breast cancer	Down	Proliferation and metastasis	^64^
circ‐0000437	CORO1C‐47aa	Endometrial cancer	Down	Microenvironment	^65^
circFNDC3B	FNDC3B‐218aa	Colorectal cancer	Down	EMT	^66^
circPGD	PGD‐219aa	Gastric cancer	Up	EMT, apoptosis	^67^
circASK1	ASK1‐272aa	Lung adenocarcinoma	Down	Apoptosis, chemoresistance	^68^
circDIDO1	DIDO1‐529aa	Gastric cancer	Down	Apoptosis	^69^
circMAP3K4	MAP3K4‐455aa	Hepatocellular carcinoma	Up	Apoptosis, chemoresistance	^70^
circMRPS35	MRPS35‐168aa	Hepatocellular carcinoma	Up	Apoptosis, chemoresistance	^71^
circATG4B	ATG4B‐222aa	Colorectal cancer	Up	Autophagy, chemoresistance	^72^
circGSPT1	GSPT1‐238aa	Gastric cancer	Down	Autophagy	^73^
circZKSCAN1	circZKSaa	Hepatocellular carcinoma	Down	Chemoresistance	^74^

CircRNA‐encoded proteins with decreased expression in tumour cells often produce effects on tumour cell proliferation that are opposite those of proteins encoded by highly expressed circRNAs. In one study, circMAPK1 expression was found to be decreased in gastric cancer tissues, and it exerted an inhibitory effect on cell proliferation by encoding a novel protein, MAPK1‐109aa, which acted on the Ras/Raf/MEK/MAPK signalling pathway. Mechanistically, MAPK1‐109aa negatively regulates the protumorigenic effects of the MAPK pathway by competitively binding to MEK1 and inhibiting MAPK1 phosphorylation, thereby inhibiting the activation of downstream factors of the MAPK1 pathway.^60^ Similarly, Wang et al. found that circMAPK14 was expressed at low levels in colorectal cancer tissues, and its encoded protein MAPK14‐175aa, which reduced the phosphorylation of MAPK14 by competitively binding MKK6, inhibited MAPK14 nuclear translocation, and induced the ubiquitination‐related degradation of FOXC1, which depends on MAPK14 blockade. The increased ubiquitination‐mediated degradation of FOXC1 inhibited the downstream signalling pathway, thereby suppressing tumour proliferation and metastasis. The degradation of FOXC1 also reversed the reduction in circMAPK14 circularization efficiency that had been blocked by FOXC1‐mediated inhibition of U2AF2 transcription, ultimately creating a positive feedback loop that promotes the expression of circMAPK14. circMAPK14 expression and MAPK14‐175aa production further enhanced the inhibitory effect of MAPK14‐175aa on the proliferation and metastasis of colon cancer cells.^61^ Liang's team found that circPLCE1 was also expressed at low levels in colorectal cancer cells and inhibited the proliferation and metastasis of colorectal cancer cells by encoding the PLCE1‐411aa protein. Mechanistically, circPLCE1‐411aa binds to the HSP90α/RPS3 complex and promotes the dissociation of RPS3, an important regulator of NF‐κB, from the complex, leading to ubiquitin‐dependent degradation of RPS3 and ultimately inhibiting NF‐κB signalling, thereby suppressing the proliferation and metastasis of colorectal cancer cells. Another recent paper reported that circSEMA4B, which is expressed at low levels in breast cancer tissues, inhibits the proliferation and metastasis of breast cancer and revealed through experimental studies that circSEMA4B exerts its biological effect not only by sponging miR‐330‐3p but also by encoding a novel protein, SEMA4B‐211aa. SEMA4B‐211aa inhibits the production of PIP3 by competing with p110 for p85 binding, thereby inhibiting the phosphorylation of the Thr308 site of AKT and ultimately negatively regulating the PI3K/AKT signalling pathway, thereby inhibiting breast cancer progression.^64^ The circAKT‐encoded protein AKT3‐174aa, circHEATR5B‐encoded protein HEATR5B‐881aa, circSHPRH‐encoded protein SHPRH‐146aa, circPINTexon2‐encoded protein PINT‐87aa and circFBXW7‐encoded protein FBXW7‐185aa were also found to function as inhibitors of tumour progression, and their expression was decreased in tumours(Table 1), (Figure 2E–H).

### 5.2 CircRNA‐encoded proteins regulate tumour cell metastasis by regulating epithelial‐mesenchymal transition (EMT)

**FIGURE 3:**
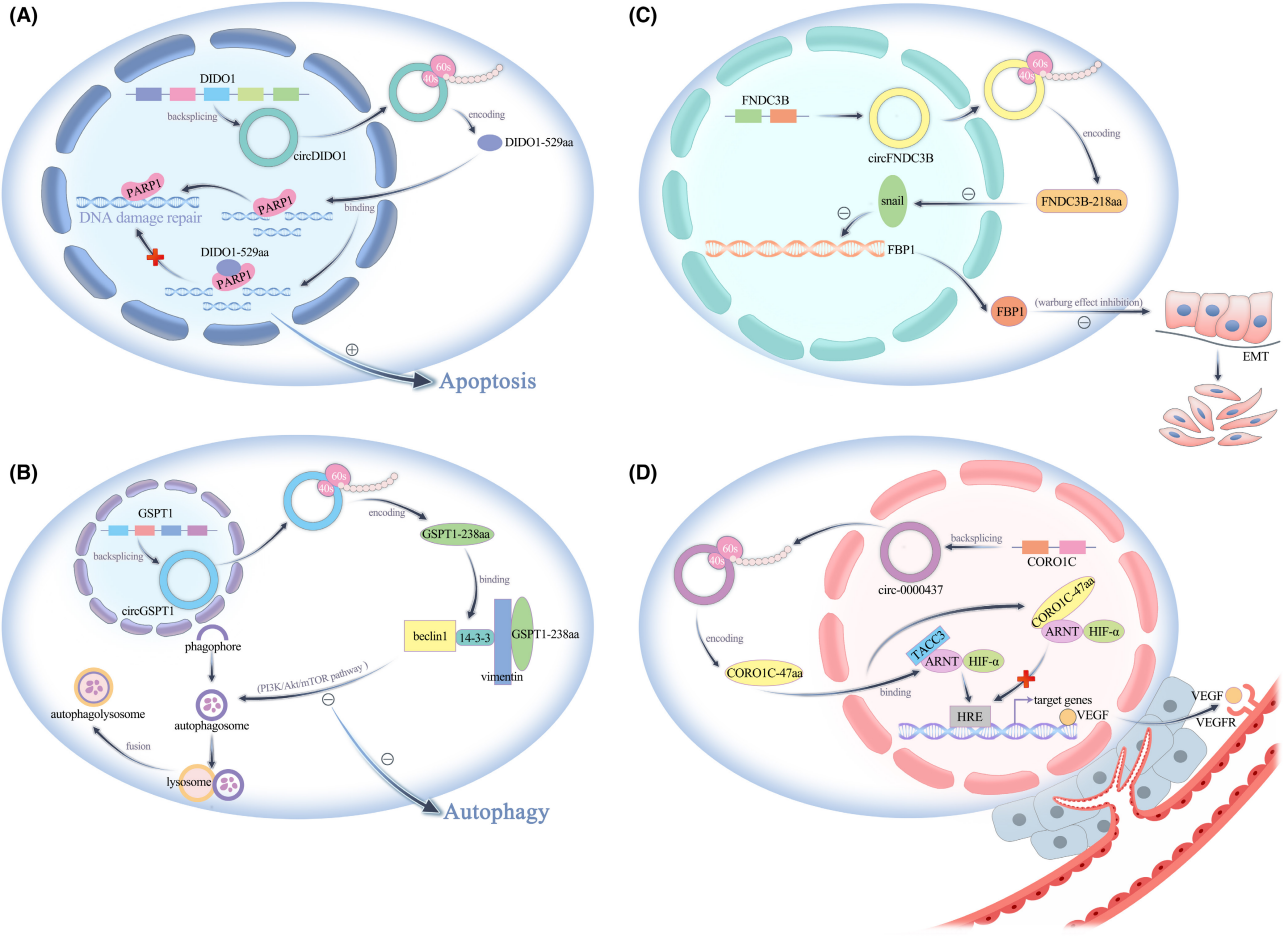
(A) circDIDO1‐529aa promotes tumour apoptosis. (B) circGSPT1‐238aa inhibits tumour autophagy. (C) circFNDC3B‐218aa inhibits tumour EMT. (D) CORO1C‐47aa affects the tumour microenvironment by suppressing tumour angiogenesis.

Pan et al. found that circFNDC3B expression was decreased in colon cancer tissues, and it inhibited EMT in colon cancer cells by encoding a novel protein, FNDC3B‐218aa. Mechanistically, FNDC3B‐218aa exerted its tumour suppressive effect by acting on the Snail/FBP1/EMT axis, and FNDC3B‐218aa suppressed Snail expression, subsequently upregulating FBP1 and inhibiting EMT in colon cancer cells. Fructose‐1,6‐bisphosphatase 1 (FBP1), one of the restriction enzymes associated with gluconeogenesis, plays an important role in glucose metabolism, while the metabolic shift affects the course of EMT, thus promoting tumour malignancy and migration, especially the Warburg effect of diminished oxidative phosphorylation and enhanced glycolysis. It is generally believed that tumour cells can evade the normal apoptotic programme and enhance their proliferation and migration ability through this abnormal glycometabolism process. This particular glycolytic behaviour not only provides nutrition for cancer cells but also makes the tumour environment more acidic, leading to extracellular matrix destruction and thus inducing tumour metastasis.^66^ In contrast, Liu's team found that high expression of circPGD in gastric cancer tissues promoted EMT and inhibited the apoptosis of gastric cancer cells. Moreover, circPGD has been shown to exert biological effects not only by sponging miR‐16‐5p but also by encoding a novel protein, PGD‐219aa, which promotes gastric cancer progression by acting on the SMAD2/3 and YAP signalling pathways^67^ (Figure 3C).

### 5.3 CircRNA‐encoded proteins regulate tumour cell proliferation by regulating the tumour microenvironment

A study revealed that hsa‐circ‐0000437 expression was decreased in endometrial cancer tissues versus normal tissues. hsa‐circ‐0000437 has been found to function as an inhibitor of neovascularization in tumour tissues by encoding a novel protein, CORO1C‐47aa. Vascular endothelial growth factor (VEGF) is an angiogenic factor that plays a key role in promoting endothelial cell proliferation and migration and increasing the permeability of tumour‐associated vessels by binding to VEGFR. TACC3 promotes the recruitment of ARNT protein at the HRE site and promotes VEGF gene expression. Immunoprecipitation (IP)‐MS studies with CORO1C47aa‐FLAG‐overexpressing cells revealed ARNT as the only common interacting factor of CORO1C‐47aa, and the enrichment of TACC3 was significantly reduced in CORO1C‐47aa‐overexpressing cells. This mechanism of CORO1C‐47aa blocked the interaction between ARNT and TACC3, leading to reduced VEGF expression and secretion, ultimately inhibiting tumour angiogenesis. In conclusion, CORO1C‐47aa affects the tumour microenvironment by blocking the blood supply to tumour tissues^65^ (Figure 3D).

### 5.4 CircRNA‐encoded proteins regulate apoptosis to interfere with tumour cell death

Zhang's team found that circDIDO1 expression was decreased in gastric cancer tissues and that it inhibited apoptosis. Their experimental study revealed that circDIDO1 exerted its biological function by encoding a novel protein, DIDO1‐529aa. Poly ADP‐ribose polymerase 1 (PARP‐1) recognizes single‐strand breaks in DNA and repairs them, and inhibition of PARP‐1 leads to decreased DNA damage repair and causes apoptosis in cancer cells. circDIDO1 encodes the DIDO1‐529aa protein, which interacts with and inhibits the activity of PARP1, which is closely related to apoptosis, thereby suppressing the DNA repair ability of PARP1, and ultimately promoting the apoptosis of gastric cancer cells^69^ (Figure 3A).

### 5.5 CircRNA‐encoded proteins regulate autophagy to intervene in tumour cell survival

Autophagy is an orderly process of intracellular self‐digestion, a biological function that enables cells to overcome nutrient deficiency and facilitates tumour cell survival and early stage tumour development. Hu et al. found that circGSPT1, which is downregulated in gastric cancer tissues versus normal tissues, inhibited autophagy in gastric cancer cells by encoding a novel protein, GSPT1‐238aa. Autophagy can be regulated by the PI3K/Akt/mTOR signalling pathway, which in turn is regulated by circGSPT1. Class III PI3Ks in the PI3K enzyme family regulate autophagic vesicle formation by forming a complex with Beclin‐1. Structural domains in PI3K interact with members of the GTPase family, and GSPT1 also acts as a GTPase and carries the same GTPase structural domain as GSPT1‐238aa. GSPT1‐238aa can interact with the PI3K/AKT/mTOR pathway via this GTPase structural domain. Akt‐mediated phosphorylation of Beclin‐1 enhances the interaction of Beclin‐1 with 14‐3‐3 and Vimentin intermediate filament proteins, resulting in the formation of an inhibitory complex including Vimentin, Beclin‐1, and 14‐3‐3 that is involved in autophagy^73^ (Figure 3B).

### 5.6 CircRNA‐encoded proteins generally regulate chemoresistance of tumour cell by regulating apoptosis and autophagy

**FIGURE 4:**
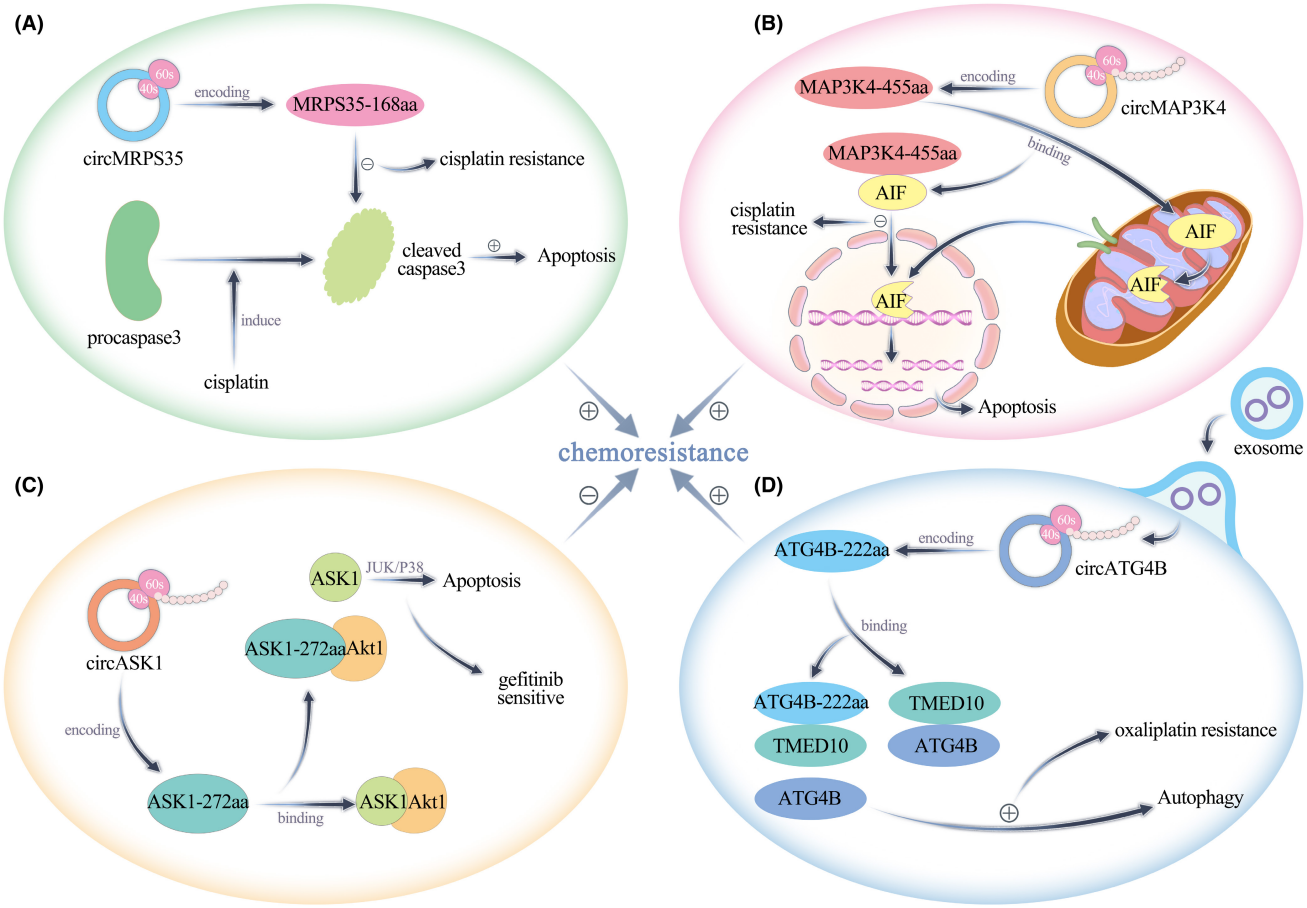
(A, B) circMRPS35‐168aa and circMAP3K4‐455aa promote tumour chemoresistance by inhibiting apoptosis. (C) circASK1‐272aa inhibits tumour chemoresistance by promoting apoptosis. (D) circATG4B‐222aa promotes tumour chemoresistance by promoting autophagy.

Chemoresistance in tumour tissue manifests as inhibition of tumour cell apoptosis. A study reported that circASK1 expression was decreased in gefitinib‐resistant lung adenocarcinoma cells and that its reduced expression played a role in enhancing the gefitinib sensitivity of lung adenocarcinoma cells by activating the ASK1/JUK/p38 signalling pathway mediated through the encoded protein ASK‐272aa. Mechanistically, ASK‐272aa competitively binds Akt1 with ASK1 and antagonizes the Akt1‐induced phosphorylation and inactivation of ASK1, thereby activating ASK1‐induced apoptosis, which in turn leads to reduced gefitinib resistance in lung adenocarcinoma cells.^68^ Duan's team found that circMP3K4 expression was increased in hepatocellular carcinoma and that IGF2BP1 recognized the m6A site on circMAP3K4 and promoted its translation to generate circMAP3K4‐455aa. Mechanistically, circMAP3K4‐455aa protects the N‐terminus of AIF by binding to endogenous AIF in mitochondria, thereby preventing AIF cleavage and inhibiting its nuclear distribution to prevent cisplatin‐induced apoptosis of hepatocellular carcinoma cells.^70^ Another study found that circMRPS35 was highly expressed after sorafenib treatment in hepatocellular carcinoma. circMRPS35 encodes a novel protein, circMRPS35‐168aa, which is significantly upregulated by cisplatin, doxorubicin and etoposide treatment in hepatocellular carcinoma. The high expression of circMRPS35‐168aa counteracts the cisplatin‐induced conversion of Caspase‐3 to cleaved Caspase‐3, which plays a key role in apoptosis and induces cisplatin resistance^71^ (Figure 4A–C).

Chemoresistance in tumour tissues can also be realized by autophagy promotion in tumour cells. Pan et al found that circATG4B expression was increased in colorectal cancer tissues. Through experimental studies, they found that circATG4B promoted autophagy in colorectal cancer cells by encoding a novel protein, ATG4B‐222aa, which increased the resistance of colorectal cancer cells to oxaliplatin. TMED10 inhibited the downstream pathway by binding to the ATG4B protease in the autophagic pathway and reduced the cleavage of the LC3 protein by free ATG4B. Further studies revealed that circATG4B was highly expressed in the exosomes of oxaliplatin‐resistant colorectal cancer cells and that it was transferred to other oxaliplatin‐sensitive colorectal cancer cells via these exosomes. circATG4B encodes the protein ATG4B‐222aa, which is a decoy to prevent ATG4B from competitively binding TMED10, thereby allowing an increased amount of ATG4B to be released and accelerating autophagy, ultimately leading to increased autophagy and inducing chemoresistance in colorectal cancer cells^72^ (Figure 4D).

## 6 PROSPECTS FOR CLINICAL TREATMENT APPLICATIONS

Based on the review of the above studies, considering that circRNA‐encoded proteins play an important role in tumour progression and that most of the current research has not involved clinical application in tumour patients, perhaps we can try to improve the prognosis by using circRNA‐encoded proteins as a key biomarker to improve the early diagnosis rate of tumours or solve the problem of drug resistance in tumour chemotherapy. As reported by Tang et al, HNRNPU‐603aa encoded by circHNRNPU promotes multiple myeloma progression by regulating the bone marrow microenvironment and alternative splicing events,^53^ and this protein may be a potential diagnostic biomarker for multiple myeloma. Another recent study reported that ATG4B‐222aa encoded by circATG4B reduces the chemosensitivity of colorectal cancer cells by promoting autophagy.^72^ Since it can act as an important biomarker, if this protein can be detected during chemotherapy, it can guide changes in the treatment plan in advance during follow‐up chemotherapy. Moreover, ATG4B‐222aa can also provide a new potential therapeutic target for oxaliplatin‐resistant colorectal cancer.

We can also try to target circRNA‐encoded proteins to treat diseases in future clinical studies. For example, some circRNAs are decreasingly expressed in tumours and can be overexpressed to translate more proteins to inhibit tumour progression; for example, SEMA4B‐211aa encoded by circSEMA4B is decreasingly expressed in breast cancer and can negatively regulate the PI3K/AKT signalling pathway to inhibit breast cancer progression.^64^ In contrast, some other circRNAs have increased expression in tumours, and the production of protein can be reduced by designing specific small interfering RNAs (siRNAs) to knockdown circRNAs expression and inhibit their biological function. For example, HER2‐103aa encoded by circHER2 is increasingly expressed in breast cancer and can activate the PI3K‐AKT pathway to promote triple‐negative breast cancer progression.^47^ After years of development, the combination of nanotechnology and medicine has solved many problems of traditional medicine and has been extensively applied in medical research. Therefore, both these overexpression and knockdown operations can be carried out by loading nanoparticles with the relevant constructs for delivery.^75^, ^76^, ^77^ In the latter case, it should be noted that the circRNAs themselves have other important biological functions, and simply knocking down the circRNAs would compromise other functions, so targeting important elements of the translation process of circRNAs to only reduce the expression of the proteins they encode may be a better option; for example, interfering with the step in which the IRES binds to the eIF4G2 complex in translation initiation could be explored.^78^ This deserves a follow‐up study.

## 7 LIMITATIONS OF CURRENT STUDIES

Currently, we have generated insights into the biogenesis of circRNA‐encoded proteins and how they function in tumours. However, in the course of our review, we have identified several limitations of current studies.

Firstly, we found that previous researches not only focused too much on the mechanism and lacked applications of treatment but also tended to study the role played by a single functional mechanism of circRNAs, while a comprehensive study of multiple different functions of a circRNA in a contemporaneous and systematic manner has been lacking. Therefore, experiments to study the miRNA‐sponging function of circRNAs during a specific timeframe are needed. Moreover, whether circRNAs function by encoding proteins is unclear. Therefore, rigorous conceptualization and discussion of research approaches are needed to address the following questions: When circRNAs play a role by acting as miRNA sponges in downstream signalling pathways and affecting cell functions, is their effect achieved through a single sponging mechanism or in conjunction with multiple mechanisms, such as protein encoding and RBP‐related mechanisms? In‐depth studies are warranted to address these questions.

Second, m6A has become a focus of current research, but the study of m6A‐related factors on circRNA‐encoded proteins has not been widely and deeply explored.

Finally, RCT and iRCT may be novel mechanisms for the translation of circRNA‐encoded proteins, and whether there are other mechanisms that can regulate circRNA‐encoded proteins? This also remains to be discovered.

## 8 DISCUSSION

This review provides a detailed description of recent advances in understanding the development of human tumour diseases regulated by circRNA‐encoded proteins. Moreover, this review provides future research directions on circRNA‐encoded proteins. For example, m6A‐related factors in circRNA‐encoded proteins have not been extensively explored, and other mechanisms that regulate circRNA‐encoded proteins may await discovery, there is still a lack of comprehensive contemporaneous and systematic research on the multiple different functions of circRNAs. In addition, no specific clinical therapeutic‐related studies have been conducted for circRNA‐encoded proteins. Recent research on circRNA‐encoded proteins in various tumours has shown that circRNA‐encoded proteins exhibit great potential for use as diagnostic markers and therapeutic development in the future. With further study, these proteins can be the basis for new drugs to improve early tumour screening, address cancer chemoresistance, increase the efficacy of tumour‐targeted therapy, or even directly participate in the treatment of cancer by combining with nanomedicine, which could prolong patient survival.

## AUTHOR CONTRIBUTIONS

**Chengwei Wu:** Conceptualization (equal); writing – original draft (equal). **Song Wang:** Conceptualization (equal); writing – original draft (equal). **Tingting Cao:** Conceptualization (equal); writing – original draft (equal). **Tao Huang:** Conceptualization (equal); writing – original draft (equal). **Lishuai Xu:** Writing – review and editing (equal). **Jiawei Wang:** Writing – review and editing (equal). **Qian Li:** Writing – review and editing (equal). **Ye Wang:** Writing – review and editing (equal). **Long Qian:** Writing – review and editing (equal). **Li Xu:** Conceptualization (equal); supervision (equal); writing – review and editing (equal). **Yabin Xia:** Conceptualization (equal); supervision (equal); writing – review and editing (equal). **Xiaoxu Huang:** Conceptualization (equal); supervision (equal); writing – review and editing (equal).

## FUNDING INFORMATION

This research was funded by the National Natural Science Foundation of China (81902515) and Natural Science Research Project of Higher Education in Anhui Province (KJ2021A0857).

## CONFLICT OF INTEREST STATEMENT

The authors confirm that there are no conflicts of interest.

## ETHICS STATEMENT

Institutional Review Board Statement: N/A. Informed Consent Statement: N/A. Registry and the Registration No. of the study/trial: N/A. Animal Studies: N/A.

## Data Availability

Data sharing not applicable to this article as no datasets were generated or analysed during the current study.
